# Effect, economic and process-evaluation of a generic function focused care program for long-term care; study protocol of a multicenter cluster–randomized trial

**DOI:** 10.1186/s12912-022-00902-5

**Published:** 2022-05-19

**Authors:** Stan Vluggen, Silke Metzelthin, Valeria Lima Passos, Sandra Zwakhalen, Getty Huisman-de Waal, Janneke de Man-van Ginkel

**Affiliations:** 1 Department of Health Services Research, Maastricht University, Care and Public Health Research Institute, Duboisdomein 30, Maastricht, 6229 GT The Netherlands; 2 Living Lab in Ageing and Long-Term Care, Maastricht, The Netherlands; 3 Department of Methodology and Statistics, Maastricht University, Care and Public Health Research Institute, Maastricht, The Netherlands; 4 Radboud University Medical Center, Nijmegen, The Netherlands; 5 University Medical Center Utrecht, Utrecht, The Netherlands

**Keywords:** Function focused care, Activities of daily living, Nurses, Geriatric clients, Behavior change, Long-term care

## Abstract

**Background:**

Nurses are in a key position to stimulate older people to maximize their functional activity and independence. However, nurses still often work in a task-oriented manner and tend to take over tasks unnecessarily. It is evident to support nurses to focus on the capabilities of older people and provide care assistance only when required. Function-Focused Care (FFC) is a holistic care-philosophy aiming to support nurses to deliver care in which functioning and independence of older people is optimized. Dutch and internationally developed FFC-based interventions often lack effectiveness in changing nurses’ and client’s behavior. Process-evaluations have yielded lessons and implications resulting in the development of an advanced generic FFC-program: the ‘SELF-program’. The SELF-program aims to improve activity stimulation behavior of nurses in long-term care services, and with that optimize levels of self-reliance in activities of daily living (ADL) in geriatric clients. The innovative character of the SELF-program lies for example in the application of extended behavior change theory, its interactive nature, and tailoring its components to setting-specific elements and needs of its participants. This paper describes the outline, content and theoretical background of the SELF-program. Subsequently, this paper describes a protocol for the assessment of the program’s effect, economic and process-evaluation in a two-arm (SELF-program vs care as usual) multicenter cluster-randomized trial (CRT).

**Method:**

The proposed CRT has three objectives, including getting insight into the program’s: (1) effectiveness regarding activity stimulation behavior of nurses and self-reliance in ADL of geriatric clients, and (2) cost-effectiveness from a societal perspective including assessments of quality of life and health-care use. Measurements will take place prior to program implementation (baseline), directly after (T1), and in long-term (T2). Parallel to the CRT, a process evaluation will be conducted to provide insight into the program’s: (3) feasibility regarding implementation, mechanisms of impact and contextual factors.

**Discussion:**

The SELF-program was developed following the Medical Research Council framework, which addresses the systematic development, feasibility testing, evaluation and implementation of complex interventions. The program has been subjected to a feasibility study before and results of studies described in this protocol are expected to be available from end 2022 onwards.

**Trial-registration:**

The study is registered in the Dutch Trial Register (NL9189), as of December 22 2020.

**Supplementary Information:**

The online version contains supplementary material available at 10.1186/s12912-022-00902-5.

## Background

Worldwide, predictions indicate that due to medical advances, the number of people aged ≥ 60 will double by 2050 from 12 to 22% [1, 2]. In the Netherlands, a similar increase is expected as the number of people aged ≥ 65 will increase most by 2060 and will account for one fourth of the total national population [3]. Although the aging of humanity is an optimistic development, it posits challenges for society, financial resources and a serious threat to people’s functional ability and independency [4–6]. Many people reach a point where formal care support to meet basic needs and complete activities of daily living (ADL) becomes inevitable. A significant goal and priority of the World Health Organization, and of national and local authorities worldwide, is to strengthen long-term care services and maximize the functional abilities and independence of older people [7, 8].

In the Netherlands, formal care support is provided by nursing care professionals and allied health professionals such as a physiotherapist en occupational therapist, throughout the entire care continuum [9–12]. For example, frail older people living at home or those residing in nursing homes are supported in ADLs such as personal hygiene and dressing, toileting, and nourishment [13, 14]. Given their direct and frequent contact, nurses are in a key position to stimulate and enable older people to maximize their activity, functional ability and independence. Despite their key role, nurses still often seem to work in a task-oriented manner and to – although well intended – take over tasks from clients unnecessarily [15]. For instance, recent observational work of den Ouden and colleagues showed that nurses took over ADLs in almost half of the cases and that nursing home residents remained inactive in over 50% of ADLs [16]. This is likely to deprive older people’s remaining abilities, impair their dignity and quality of life, and may ultimately lead to disability [17–19]. Although nurses generally do acknowledge their potential active role in promoting activity and self-perceive to have sufficient knowledge and recognize the benefits not only for their clients but also for themselves, various barriers seem to impede them to adequately support and enable older people to optimize their daily functioning [20–22]. For example, barriers may occur at the level of the client (e.g., lack of knowledge), the care worker (e.g., lack skills), the environment (e.g., narrow hallways), and the organization (e.g., lack of policy and support) [20, 22, 23]. For this reason, it is evident to foster programs that support nurses to adopt a proactive care attitude, to focus on the capabilities of older people, and to provide care support only when required.

Function-Focused Care (FFC) is a holistic care-philosophy that aims to support nurses to deliver care in which daily functioning and independence of clients is optimized. The key principle of FFC is that nurses are deemed to change from doing things ‘for’ the client, to encouraging clients to engage in functional and physical activity during all care interactions, taking into account their capabilities [19]. The principles of FFC-philosophy have guided the development of various (inter)national interventions for various care settings [24]. In general, such interventions have shown to be feasible in practice but have demonstrated mixed results regarding their effectiveness in improving care professionals’ activity stimulation behavior, and clients’ engagement in functional and physical activity [25].

Over the past decade, the Netherlands has built a considerable record regarding the systematic development of interventions based on the FFC- or related care philosophies in both long-term and acute care settings. Guided by the Medical Research Council (MRC) framework for complex interventions [26], divergent FFC-based interventions were developed, implemented and evaluated in Dutch long-term and acute care settings [27–30]. In line with internationally developed interventions, Dutch FFC-based programs showed equivocal results and thorough evaluations are suggested to improve future programs [25].

Syntheses from Dutch practices have provided valuable lessons learned and implications to optimize future programs and develop an advanced FFC-program, including: 1) the development of a generic FFC-program applicable to a variety of nursing care settings, which allows for tailoring to setting specific elements and needs of its participants, 2) addressing all FFC-components jointly instead of single elements, 3) the inclusion of a comprehensive interactive training component that provides ownership to its participants, 4) the incorporation of an extended integrated theory of behavior change, compared to the one addressed in the current philosophy, and 5) improvement of managerial support by ensuring sufficient time and staff resources [31]. These implications have led to the development of a renewed and advanced generic FFC-program, applicable to a variety of long-term care settings; the ‘SELF’-program. The program has recently been subjected to a feasibility and acceptability study in nursing home care, which yielded useful input from nurses for pre-implementation improvements.

The aim of the current paper is twofold: first the outline, content and theoretical background of the newly developed SELF-program are presented. Subsequently, we describe a comprehensive protocol for the assessment of the program’s effect, economic and process-evaluation among nurses and geriatric clients in a multicenter cluster-randomized trial.

## Methods and design

### SELF-progtram: outline and content

‘SELF’ is a Dutch acronym for *self-reliance, autonomy, life quality and functionality.* The ‘SELF’-program is a multi-component program based on the principles of Function Focused Care. The FFC-philosophy assumes a holistic approach in changing nurses’ care and consequently clients’ activity behavior, including (1) an assessment and revision of organizational policies and environment, (2) education of nurses, (3) the establishment of FFC-goals for clients, and (4) continued motivation and mentoring of nurses and clients [24].

The SELF-program is an embodiment of the FFC-philosophy and its components, and addresses all components in conjunction, including a comprehensive interactive training program as its core component. In addition, the SELF-program takes into account the lessons learned and implications from previous practices, meaning that SELF is applicable to a variety of nursing care settings, allows for setting- and context specific tailoring and needs of its participants, incorporates extended integrated theory of behavior change, and improves managerial support. The ultimate aim of the SELF-program is to improve activity stimulation behavior of nurses in long-term care services, and with that optimize levels of self-reliance in ADL in geriatric clients. The SELF-program is theoretically grounded in the Integrated Change Model for explaining and changing health-related behavior [32].

### Theoretical background

Nurses are deemed to change their own daily care behavior towards stimulating and enabling clients to – in turn – optimize their active involvement and self-reliance in ADL. Therefore, a sequential dual behavior change process is incorporated in the SELF-program. The dual sequential behavior change process forms the basis throughout the SELF-program and intends to primarily increase awareness, motivation and willingness in nurses to stimulate clients, where after nurses are trained to improve client’s activity behavior.

**Fig. 1:**
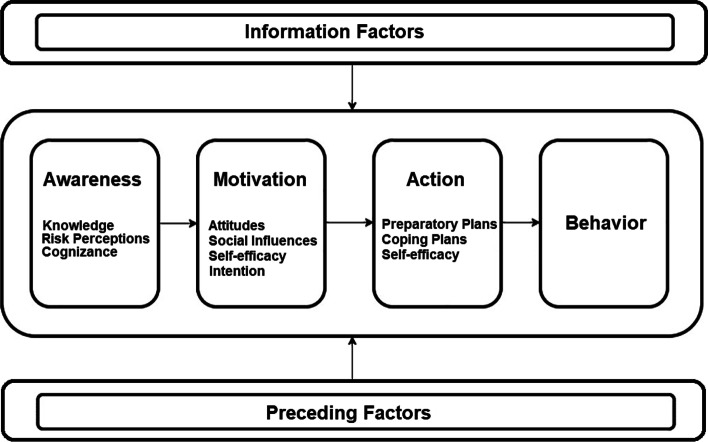
The integrated change model [32]

The FFC-philosophy assumes that in order to improve nurses’ stimulation behavior, one’s self-efficacy and outcome expectations of the desired behavior need to be enhanced [24]. While in fact these are two well-known determinants of a deliberate process of behavior change, integrative models of behavior change such as the Integrated Change Model (ICM), and implications from recent work suggest to address a broader spectrum of behavior change determinants and behavior change phases [32]. The ICM (Fig. 1) assumes that behavior change is not merely the result of being confident in one’s own abilities and viewing positive outcome expectations. By integrating various well-known socio-cognitive theories [33, 34], and building on the core principles of the Theory of Planned Behaviour [35], the model assumes a phased behavior change process differentiating between an awareness, a motivation and an action-planning phase, influenced by information and preceding factors.

The model assumes that behavior change is a result of becoming aware of its necessity by activating risk perceptions and increasing knowledge of the desired behavior and its consequences. Moreover, a person’s cognizance level indicates if a person is (in)correctly aware of carrying out the desired behavior. From a behavior change perspective, creating awareness is an important prerequisite of enacting a behavior [36]. Subsequently, if sufficient awareness of behavior change is present, a weighing of the pros and cons (outcome expectations) of the desired behavior, perceptions of social influences, and the level of one’s own belief to successfully carry out the desired behavior (self-efficacy), determines the motivation to change someone holds. The strength of one’s intention or willingness to change a behavior is determined by these awareness and motivational factors. It is well known that expressing a high intention towards behavior change does not necessarily guarantee successful behavior change [37]. People who express a high intention towards behavior change have a higher likelihood of successful translation of this intention into practice, by performing action planning: preparing the behavior adequately and anticipating challenging situations by the formulation of coping plans. In this phase, self-efficacy again plays a key role in enacting these plans.

The action phase in the behavior change process of nurses overlaps with the cycle of behavioral change in their clients. To illustrate, once nurses establish a willingness to change, the action phase includes raising awareness, and motivating and enabling clients to optimize their self-reliance and time spend in ADL. However, the behavior change process for geriatric clients in the training component of the SELF-program is rather a mix between rational behavior change methods such as assumed in the ICM, and more unconscious and practical strategies, such as providing verbal cues [38]. This is so, because Dutch long-term care services include a mixture of geriatric clients with different somatic and psychogeriatric backgrounds, for which the combination of rational as well as unconscious behavior change methods may prove effective in enhancing self-reliance in functional activity more than rational methods alone.

### Outline of the SELF-program

First, the policy of the organization is assessed and if necessary reviewed whether the organization aims to provide care in which daily functioning and independence of frail older people is maximized. In case of absence of organizational policy, the SELF-program offers an example policy document to be discussed with and supplemented by the organizational board and team managers [24]. In line with recommendations of previous work, the SELF-program ensures that the policy is not only in place, but also adequately and repeatedly propagated to the nurses, e.g,. within the training component or team meetings [31]. Last, managerial visibility and support is encouraged to reinforce the desired behavior change among nurses.

**Fig. 2:**
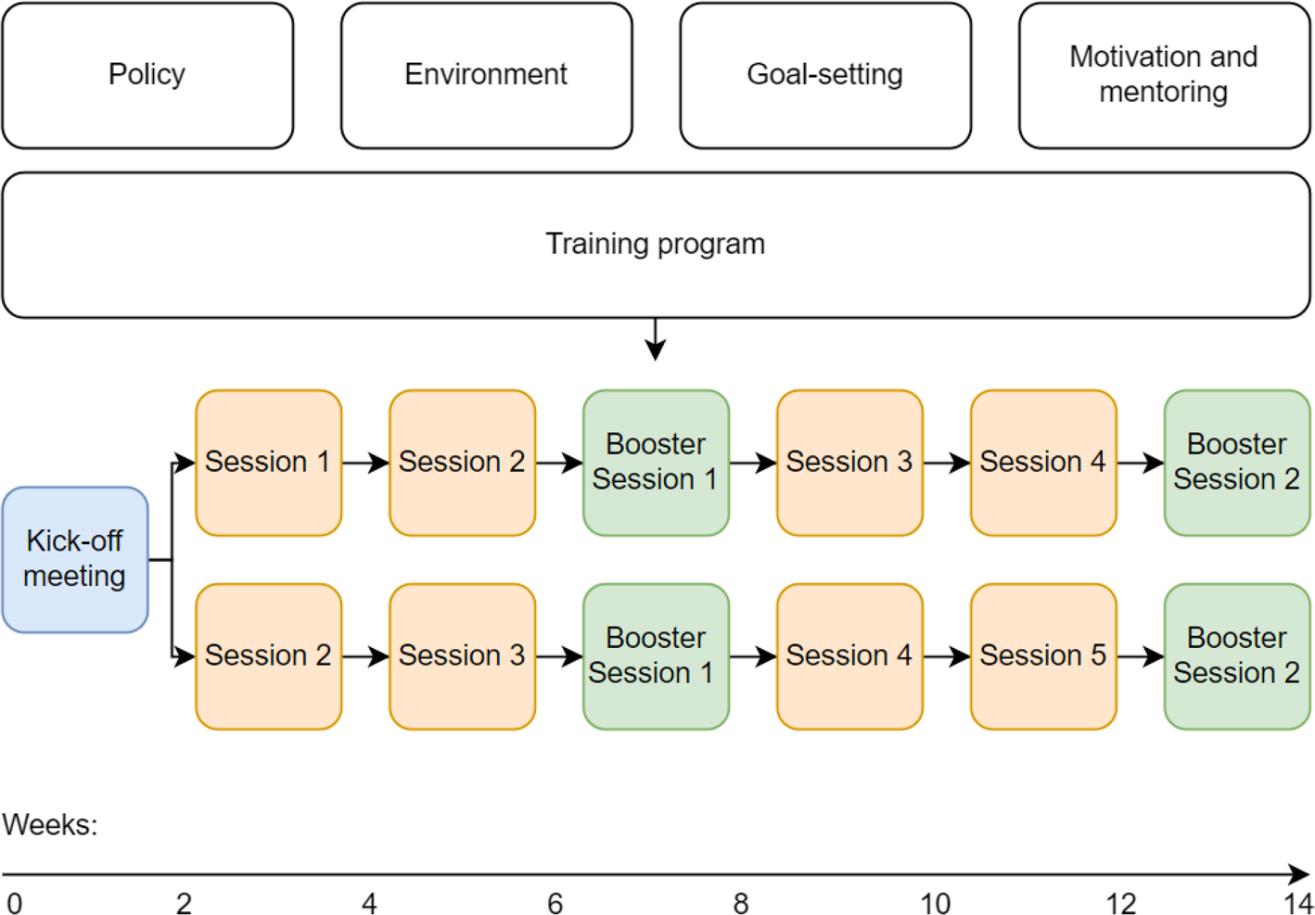
Schematic overview of the SELF-program

The key component of the SELF-program is a comprehensive tailor-made training program, as staff training is considered a key-element in the desired change in day-to-day clinical behavior or nurses [22]. The training program consists of seven face-to-face sessions varying from one to two hours, spread over a period of three months, see Fig. 2. Each session consists of several interactive assignments, with a strong focus on bottom-up learning and autonomy to its participants. For instance, assignments are conducted in small groups with a subsequent plenary discussion. Sessions are guided by a trainer appointed from the organization, for example someone with a background in education or quality improvement. In addition, an assistant-trainer is appointed on a voluntary basis from the concerned care-team to provide the main trainer with adequate examples from daily care practice and to support the trainer in the conduct of the training sessions.

Within the training program, the core components of the FFC-philosophy are addressed as well as an empathic focus on behavior change in nurses towards enhanced activity stimulation behavior. The training program starts with a two-hour kick-off session intended to increase awareness of activity stimulation behavior in nurses and to address and propagate the concerned organizational policy towards self-reliance and independence of clients. Predominantly, the kick-off session aims to map the starting point of the care team with regard to the willingness to improve their activity stimulation behavior. This way, the subsequent educational trajectory can be tailored to the needs from the care team in question. Based on triangulation, i.e., taking into account perspectives of the trainer, the manager of the care team and nurses themselves, a decision is made whether to continue with session 1 or 2 following the kick-off session. In case the team is insufficiently aware, motivated and willing to improve their function focused care behavior, the team will continue with session 1. On the contrary, if the team shows sufficient awareness of the necessity to change and willingness to improve their FFC-behavior, the team continues with session 2. In session 1, participants discuss awareness, attitudes and the necessity for behavior change towards enhanced activity stimulation behavior. Session 2 builds on session 1 by covering the topic of motivation and willingness to change behavior, as well as the essence of a consistent application of the intended behavior among care team members. Last, in session 2, one or more nurse champions (practice coaches) are appointed to ensure the intended behavior is adequately implemented in practice and to facilitate the ongoing motivation and mentoring of staff and clients after the training program has been completed [24, 39]. Session 3 is about translating the willingness into goals and concrete actions to facilitate actual behavior change. For instance, rational and practical tools are presented and discussed to pursue an optimization of functional activity and self-reliance in geriatric clients. Nurses are encouraged to exchange their experiences in encouraging functional activity in their geriatric clients. Compared to all other sessions, booster session 1 (midway) and booster session 2 (at the end) both last 1 h. Booster session 1 reviews the environment geriatric clients reside in, either in home care of in nursing homes and addresses viable options for adapting the environment. This element is supported by a checklist addressing relevant problem areas and potential environmental changes to optimize engagement in physical and functional activity, based on Resnick and colleagues [24]. Both booster sessions also intend to recap the content of the previous sessions to assure the nursing care team is actually ready to proceed to subsequent sessions. Session 4 is an actor-guided role-play session in which an external actor with expertise in healthcare is hired to practice activity stimulation behavior in a real-life and interactive manner. In session 5, attention is payed to behavior maintenance through continued motivation and mentoring of nurses and clients and by establishing concrete and tailored strategies and goals per client to encourage functional activity. Each session is concluded with a practical assignment to be discussed in the subsequent session.

### Study protocol for effect, economic and process-evaluation

To examine the effectiveness and cost-effectiveness of the SELF-program, a two-arm (SELF-program vs care as usual) multicenter cluster-randomized trial (CRT) will be conducted. The CRT has three objectives, including getting insight into the program’s: (1) effectiveness regarding activity stimulation behavior of nursing care professionals and in self-reliance in activities of daily living of geriatric clients (effect evaluation), (2) cost-effectiveness from a societal perspective including assessments of quality of life and health-care use (economic evaluation). Parallel to the CRT, a process evaluation will be conducted to provide insight into the program’s: (3) feasibility regarding implementation, its mechanisms of impact and contextual factors (process evaluation). The study was approved by the Medical Ethics Committee of the Zuyderland Hospital (METC-Zuyd; METCZ20210007). In line with Dutch regulation, no specific ethical approval was needed for this study according to the rules of the Medical Research Involving Human Subjects act (WMO) [40]. Amendments, if any, will again be submitted to the medical ethics committee. The study is registered in the Dutch Trial Register (NL9189). The protocol follows the evidence based recommendations addressed in the Standard Protocol Items Recommendations for Intervention Trials (SPIRIT) 2013 [41]. The SPIRIT-checklist is available as supplementary material to the trial protocol. The study recruitment commenced in spring 2021 and is expected to last until spring 2022. Data collection is expected to last until autumn 2022.

### Procedure and recruitment of participants

To examine the program’s effectiveness and cost-effectiveness, two participant groups will be recruited, i.e., nurses working in nursing home care and geriatric clients receiving nursing home care. Care organizations across the Netherlands are able to take part in the study as the SELF-program is widely disseminated through various (social) media platforms and national conferences. To be eligible to participate, organizations have to offer nursing home care. In case of organizational willingness to participate, formal approval of the board of directors will be requested and the client council will be informed about the study. In close agreement with organizational boards and team and location managers, nursing home wards will be selected to participate in the trial. Wards hosting somatic clients, as well as wards where psychogeriatric clients reside are eligible to be included in the study. Within an organization, dyads will be created consisting of two nursing home wards, which will then be pair-matched according to size and ward type, e.g., somatic or psychogeriatric ward. Of the two included wards from a dyad, one will be randomly allocated into the intervention group (SELF-program) or into the control group (care as usual), and clients residing at a ward will be allocated accordingly. Randomization will be conducted by means of computer-randomization using *random.org.* The unit of intervention will be the care ward, the unit of analysis will be the nursing care professional or the resident respectively.

To minimize the probability of contamination bias, dyads will be created of wards that do not collaborate in daily practice. In case of collaboration between wards, something likely to occur within nursing homes, randomization will take place on location level. Nursing home organizations in the Netherlands usually consist of multiple locations, which in turn consist of a varying number of wards. In the event of collaboration between wards within a location, the entire location is included in a dyad with a pair-matched comparator location. For example, a nursing home location with three collaborating psychogeriatric wards, will be pair-matched with a similar location.

### Nurses

All nurses who are part of a nursing home ward are eligible to participate. Those who are not involved in care delivery in all ADL are excluded. Before the start of the study, all nurses allocated to either the intervention or control condition will receive a non-committal information letter by email explaining study details and a link to the digitalized questionnaire of the study. After consideration, nurses will be asked whether to provide online informed consent to participate. After signing the digital informed consent form, participants are directed to the baseline questionnaire.

### Geriatric clients

Geriatric clients are eligible to participate if they are residing at the concerned ward at time of ward inclusion. Clients are excluded from participation when aged < 65 years old, or when terminally ill or bedbound. In addition, those with a temporary stay (< 6 months) are excluded. Data collection for geriatric clients will be accomplished by the primary responsible caregiver, who will be asked to fill in the questionnaires for their client(s). Depending on the level of cognitive and physical impairment, the primary responsible caregiver can fill in the questionnaire alone or together with the geriatric client. Before data-collection of geriatric clients, written informed consent will be obtained from either the client itself or a responsible party, generally a family member appointed as the primary contact person of the client.

### Data collection, primary outcome and power calculation

#### Effect evaluation

Data with regard to the effect evaluation of the SELF-program will be gathered from both nurses and geriatric clients. All data will be collected through digitalized questionnaires.

### Nurses

Since the SELF-program is directly pointed at nurses, this participant group is considered the primary target group. The primary outcome for nurses, i.e. the level of stimulation behavior of self-reliance in ADL among geriatric clients, will be assessed at baseline (before implementation of the SELF-program; T0), directly after implementation of the SELF-program (approximately 3 months after the baseline assessment; T1), and 9 months after the baseline assessment (T2). The validated self-report MAINtAIN questionnaire will be used to assess the primary outcome measure [42]. The MAINtAIN comprises three subscales with a total of 19 items, representing whether nurses stimulate clients in a variety of daily activities, that can be rated on a 9-point Likert scale, ranging from ‘never’ to ‘always’. Of the 19 items, 8 deal with encouraging ADL, 6 with encouraging instrumental ADL and 5 with encouraging general function and physical activity. The 8 items focusing on encouraging ADL will be used as a the primary outcome.

Demographic characteristics of nursing care professionals will be assessed at baseline only. These include the participants’ gender (male, female or open answer option), age, education level (low; no education up to lower technical education, medium; general secondary education up to secondary vocational education or high; school of higher general secondary education up till university degree), nursing profession (nursing aide, certified nursing assistant or registered nurse), years of working experience in nursing care and within the current care setting, and number of working hours according to contract.

### Geriatric clients

The main outcome for geriatric clients, i.e. self-reliance in daily functioning, will be assessed at baseline (before the implementation of the SELF-program for care professionals; T0), directly after implementation of the SELF-program (approximately 3 months after the baseline assessment; T1), and 6 months after the baseline (T2). The relative shorter follow-up period at T2 for geriatric clients compared to the one for nursing care professions will be applied because a higher level of turnover of geriatric clients is to be expected given the nature of the setting. The Groningen Activity Restriction Scale (GARS-4) will be used to assess the perceived self-reliance in daily functioning of geriatric clients [43]. The GARS-4 consists of two subscales with a total of 18 items that can be rated through a hierarchical 4-point answering scale ranging from ‘Yes, I can do the particular task completely independently without any effort’ to ‘No, I cannot do the particular task completely independently, only with help from others’. Of the 18 items, 11 items examine self-reliance in ADL activities and 7 items examine self-reliance in instrumental ADL activities. Only the 11 items of the ADL-subscale will be assessed given the 7 items of the instrumental ADL-subscale are less applicable to geriatric clients residing in nursing homes.

Demographics of geriatric clients will be assessed at baseline only. These include the client’ gender (male, female or open answer option), age, education level (low; no education up to lower technical education, medium; general secondary education up to secondary vocational education or high; school of higher general secondary education up till university degree), living arrangement (single, married, in a relation whether or not living together, divorced, widowed), years of receiving nursing home care, and whether and from whom the client received informal care. For both their own and their client questionnaires, nurses will be prompted periodically to complete either the baseline or follow-up questionnaires in case these are yet to be completed.

### Sample size calculation

Given the cluster-randomized trial design, the guidelines prescribed by Breukelen and Candel (2012) were applied for sample size calculation (N) [44]. The SELF-program is expected to induce a difference in groups’ averages of the primary outcome (the MAINtAIN-score), corresponding to a small to medium effect size of 0.4 (Cohen’s *d*). Further, the mean cluster size, i.e. the average number of nursing care professionals per ward, is estimated to be 15. Using the two-tailed t-test, with power and significance level of 80% and 5%, respectively, the required number of clusters (K) was then computed, using an interclass correlation coefficient of 0.05 to correct for the cluster-randomized design effect (DE), yielding K ~ 8. Subsequently, K was increased in 10% to adjust for differences cluster sizes, and the associated sample size was further augmented to account for a 20% attrition of care professionals between baseline and post-treatment. This yielded a total sample size of *N* = 330 (165 per arm) care professionals and geriatric clients in K = 22 nursing home wards, or 11 ‘dyads’. In order to achieve adequate participant enrolment, we aim to include > 22 nursing home wards.

### Economic evaluation

Data with regard to the economic evaluation of the SELF-program will be gathered from geriatric clients. Similar to the effect-evaluation, data will be collected through digitalized questionnaires client-data will be provided by the first responsible caregiver. Client-data will be gathered at the same time points as for the effect-evaluation in geriatric clients see Table 1. The economic evaluation is conducted according to the Dutch guidelines for economic evaluations in healthcare, taking into account a societal perspective [45]. Both a cost-effectiveness analysis (CEA) and cost-utility analysis (CUA) will be performed in which clinical outcomes and healthcare use and costs are assessed. In the CEA and CUA, incremental costs are compared with the incremental effects of the comparable treatments addressed in the trial (SELF-program versus care as usual) [45].

**Table 1:** Overview of data collection for effect and economic evaluation

**Outcomes**	**Measures**	**Time Points**		
**Effect evaluation**		Baseline (T0)	Follow-up 1 (T1)	Follow-up 2 (T2)
*Nursing care professionals*				
Level of stimulation behavior	MAINtAIN; (19 items)	X	3 months	9 months
*Geriatric clients*				
Self-reliance in daily functioning	GARS-4; (18 items)	X	3 months	6 months
**Economic evaluation**				
Clinical outcomes				
Self-reliance in daily functioning	GARS-4; (18 items)	X	3 months	6 months
Health-related quality of life	Euro-QOL-5D (5 items)	X	3 months	6 months
Health care use and costs				
Health care utilization	Questions derived from the iMTA Medical Consumption Questionnaire	X	3 months	6 months

### Clinical outcomes

The primary outcome for the CEA will be the degree of self-reliance in daily functioning of geriatric clients, as measured by the GARS-4 [43]. Within the CUA, the health outcome of interest is given by quality adjusted life years (QALYs), which are determined by using a generic health-related quality of life measurement instrument known as the EuroQol-5D-5L (EQ-5D-5L) [46]. The instrument, of which a Dutch tariff has been derived [47], assesses five dimensions of health-related quality of life: mobility, self-care, usual activities, pain/discomfort and anxiety/depression. Each dimension is rated on a 5-point scale ranging from ‘no problems’ to ‘extreme problems’. Ratings on the five dimensions are summed and utilities of the CUA are operationalized by QALYs [45]. Last, current health status is assessed by the Dutch version of the EuroQol visual analogue scale (EQ-VAS) [47].

### Healthcare use

Healthcare use will be measured by items derived from the iMTA Medical Consumption Questionnaire [48]. Healthcare use, as well as patient and family costs will be measured, including: 1) the number of appointments with a general practitioner, practice nurse, physiotherapist, occupational therapist, speech therapist or dietician, 2) hospital care, i.e. acute care, outpatient care or admissions, 3) informal care, and 4) medication use. The valuation of all costs is based on the updated Dutch manual for cost analyses in healthcare research, of which an updated version of 2021 will be used to express current cost-prices [49]. A schematic overview of the enrolment, interventions and assessments, according to the SPIRIT 2013 guidelines, is presented in Table 2 [41].

**Table 2:** Schematic overview of enrolment, interventions and assessments

	**STUDY PERIOD**				
	**Enrolment**	**Allocation**	**Post-allocation**		**Close-out **^**a**^
**TIMEPOINT**	***-t***_***1***_	**0**	***t***_***0***_	***t***_***1***_	***t***_***2***_
**ENROLMENT:**					
**Eligibility screen**	X				
**Informed consent**	X				
**Allocation**		X			
**INTERVENTIONS:**					
***SELF-program***			X		
***Care as usual***			X	X	X
**ASSESSMENTS:**					
**Nurses:**					
*** Demographic variables***		X			
*** MAINtAIN questionnaire***		X		X	X
**ASSESSMENTS:**					
**Geriatric clients:**					
*** Demographic variables***		X			
*** GARS-4 questionnaire***		X		X	X
*** EQ-5D-5L questionnaire***		X		X	X
*** Economic evaluation variables***		X		X	X

^a^9 months for nursing care professionals and 6 months for geriatric clients respectively

### Process evaluation

Parallel to the effect and economic evaluation, a comprehensive process evaluation will be performed according to the guidelines of the MRC-framework [50]. The process evaluation intends to assess the feasibility of the SELF-program regarding implementation, its mechanisms of impact and contextual factors that potentially have an influence on implementation and outcomes. Process evaluations of complex interventions usually consist of mixed-method approaches, including both qualitative and quantitative data-collection methods (focus group) interviews and questionnaires. This study applies both methods among program participants and those delivering the program, i.e. trainers.

### Implementation

Effects of an intervention may depend on the degree to which the intervention was implemented [50]. Commonly, process evaluations investigate a number of implementation parameters to draw reliable conclusions about a program’s effectiveness, including fidelity (whether the intervention was delivered according to plan), dose delivered (quantity of the intervention delivered), dose satisfaction (quantitative and qualitative evaluation of satisfaction with the intervention), adaptations (adaptations made to the intervention during implementation), and reach (whether and to what extent the intended target audience is exposed to the intervention). Data on implementation will be collected from various sources, through different data-collection methods, i.e. questionnaires, (attendance) logbooks, focus group interviews, and checklists, and on various time points of which before, during and after implementation (see Table 3).

**Table 3:** Overview of data collection for process evaluation

Process evaluation component	Source	Data-collection method	Time points
**Implementation**			
*Fidelity*			
Delivery according to plan	Trainers	Checklists and focus group interviews	During and after implementation
*Dose delivered*			
Quantity of delivery	Trainers	Checklists and focus group interviews	During and after implementation
*Dose satisfaction*			
Satisfaction intervention (delivery)	Interventionists	Questionnaire and focus group interviews	During and after implementation
*Adaptations*			
Alterations made during implementation	Trainers	Logbook and focus group interviews	During and after implementation
*Reach*			
Extent to which target group was exposed to intervention	Interventionists	Attendance logbook and focus group interviews	During and after implementation
**Mechanisms of impact**			
Mechanisms assumed to produce change in outcome behavior	Interventionists	Questionnaire (31 items) Focus groupinterviews	Baseline, 3 months and 9 months after baseline
**Contextual factors**			
Barriers and facilitating factors that may influence implementation and outcomes	Trainers and Interventionists	Logbook and focus group interviews	During and after implementation

### Mechanisms of impact

An exploration of the mechanisms through which interventions impact the desired behavior change is pivotal to understand how such effects occurred [51]. The primary outcome of the SELF-program is the level of stimulation behavior of self-reliance in activities of daily living among geriatric clients. In its core, the Integrated Change Model, applied as theoretical background to the SELF-program, assumes that a particular behavior is predicted from personal beliefs related to ones attitudes, perceptions of social influence, perceived self-efficacy in challenging situations, and intention [32]. A total of 31 mechanisms of impact questions will be addressed: attitudes will be measured with 11 items, perceptions of social influence with 8 items, and intention with a single item, which all can be rated on a 5-point Likert scale ranging from ‘totally disagree’ to ‘totally agree’. Self-efficacy will be measured with 11 items that can be rated on a 5-point Likert scale, ranging from ‘no confidence’ to ‘total confidence. Items are derived from previous work on beliefs of stimulation behavior, and previous applications of the Integrated Change Model [32, 52].

### Contextual factors

External factors may act as a barrier or facilitating factor to the implementation of an intervention and its outcomes [50]. Understanding the context in which an intervention is implemented is important to interpret findings and generalize results beyond it. Therefore, a logbook will be kept on those factors acting as barriers or facilitators to the implementation and outcomes of the SELF-program. In addition, post-implementation focus group interviews with interventionists and trainers will discuss relevant contextual factors and suggestions for improving the SELF-program. Table 3 presents an overview of the data-collection of the process-evaluation.

### Data analyses

#### Effect evaluation

Summary statistics of central tendency (mean, median) and dispersion (standard deviation, interquartile range) will be used for continuous variables, as appropriate, to describe the two groups, whereas categorical variables will be summarized by absolute count and relative percentages (%).

Effect evaluation will be conducted according to the intention-to-treat principle with mixed (multilevel) linear regression analyses to accommodate the hierarchical structure of the data: measurements are nested within individuals, which are nested within a ward. Both wards and subjects will constitute the random effects (three-level models). Treatment, time and, their interaction, capturing treatment vs. control average differences over time, will make up the relevant fixed-effects, together with background characteristics that will be included in the model as covariates. Outcomes will be standardized to be interpreted as effect sizes (Cohen’s *d*). The regression coefficient of the treatment*time interaction in the mixed linear regression analysis can, therefore, be interpreted as Cohen’s *d*. To establish which factors are linked to (a possible) differential attrition between the groups, a logistic regression analysis with be conducted. SPSS for Windows, version 27 will be used for all statistical analyses. The level of statistical significance will be set at 0.05 (using two-tailed tests). In case of significant interactions, subgroup analyses will be conducted to determine the differential effects. In case of unintended effects due to the intervention or due to the trial conduct, these will be reported accordingly.

#### Economic evaluation

The economic evaluation will evaluate the cost-effectiveness of the SELF-program compared with care as usual over 6 months. The principal analysis will be the incremental cost per quality adjusted life year gained from the societal perspective using the EQ-5D-5L to calculate QALYs as recommended by the Dutch Healthcare Institute [45]. This will be calculated as the mean cost difference between the SELF-program and care as usual divided by the mean QALY difference to give the incremental cost-effectiveness ratio (ICER). Use of QALYs as a generic health outcome measure allows policymakers to compare cost-effectiveness results across different sectors to guide resource allocation decisions. Unit costs will be taken from the Dutch Manual for Costing in Economic Evaluations [49] and other standard published sources. The cost of the SELF-program will also include the costs related to the external actor, who is hired to guide the role-play session.

Estimates for mean total costs and QALYs’ incremental between the SELF-program compared with care as usual over 6 months will be derived using regression analysis to control for differences in baseline utilities and costs, as well as relevant stratification variables. A secondary analysis will include the cost per point change in the primary outcome measure (GARS-4) gained of the SELF-program compared with care as usual over 6 months. Where necessary, analyses will take into account clustering at the nursing home care level via random intercept models. Regression analysis will also be used in the secondary cost-effectiveness analysis to control for potential imbalances in baseline GARS-4 values and stratification variables. To quantify uncertainty around cost-effectiveness results, non-parametric bootstrap methods will be used and 95% bias-corrected and accelerated bootstrap confidence intervals will be calculated for the mean total cost and QALY/GARS-4 incremental estimates. The bootstrap replications will also be used to construct cost-effectiveness planes and cost-effectiveness acceptability curves, from which the probability that SELF-program is cost-effective compared to care as usual at 6 months of the trial will be computed for a range of values of the willingness to pay thresholds based on current recommendations [45].

In the event of considerable proportions of missing economic outcome data, the use of multiple imputation techniques will be explored to handle missingness. We will also subject the results to additional sensitivity analyses. For example, we will explore the cost-effectiveness and cost-utility of SELF-program compared with care as usual based on: a health and social services cost-perspective (including only healthcare costs); the complete cases (participants with complete data for all outcomes).

### Process-evaluation

Both quantitative and qualitative methods will be used to analyze the data obtained from the process evaluation. Quantitative data on reach, dose delivered, dose satisfaction and mechanisms of impact will be analyzed using descriptive and comparative statistics using SPSS software. Qualitative data obtained from the focus group interviews will be transcribed verbatim, anonymized and analyzed using Nvivo Software [53] using a predefined thematic coding scheme related to concepts addressed in the process. We aim to analyze the process evaluation data prior to the effectiveness and cost-effectiveness outcomes in order to prevent biases in interpretations of results [50].

## Discussion

The SELF-program is a nursing program based on the principles of Function Focused Care, and aims to improve activity stimulation behavior of nursing care professionals in long-term care services, and with that optimize levels of self-reliance in ADL in geriatric clients. The intended cluster-randomized trial in nursing home care described in this paper, aims to provide insight into the program’s effectiveness and cost-effectiveness, and is supplemented with a thorough process-evaluation to examine the program’s feasibility regarding implementation, its mechanisms of impact and contextual factors.

Recent reviews show that current FFC-based programs across various care settings show mixed effects on improving either care professionals’ FFC-enhancing behavior and clients’ engagement in physical and functional activity [25, 54]. Based on suggestions from these reviews and synthesized findings from process evaluations of the previously developed and tested Dutch FFC-based programs, several lessons and implications have been formulated to optimize future programs and develop an advanced generic FFC-program (SELF) accordingly [31]. In brief, these implications include: 1) the development of a program applicable to a variety of care settings which allows for setting specific tailoring, 2) addressing all FFC-components in conjunction, 3) incorporating a comprehensive interactive educational component, 4) incorporating of an extended integrated theory of behavior change, and 5) improvement of managerial support by ensuring sufficient time and staff resources.

A key principle of FFC-based programs is the holistic approach in changing nurses’ care and consequently clients’ activity behavior [24]. Although current Dutch programs are based on the principles of the FFC-philosophy or similar care concepts, not all aspects considered relevant are addressed in conjunction. Multicomponent interventions are considered most promising in enhancing the functional abilities of clients [54], thereby building on a comprehensive and interactive educational component as a core element to facilitate staff training [55]. To address the uniformity, which seems to be lacking given the wide variety of available interventions nationally and internationally, a generic function focused care program was developed to be applicable in long-term care. This generic nature does not imply the absence of setting-specific tailoring or tailoring to the needs of participants. In fact, a key implication addressed in the SELF-program is to tailor the content to the setting the program is to be carried out in, e.g. home care, or the knowledge level and willingness of participants to practice FFC within the framework of the developed generic approach [56]. Tailored and interactive educational strategies, including motivational and encouraging techniques, are considered promising interventions when aiming for geriatric clients to become more empowered [57]. This way, the demand for developing more uniform interventions is met – given FFC-programs respond to a topic that is relevant in all nursing care settings – as well as need to create greater cognitive processing in participants by the application of tailoring [58].

This study set up has several strengths. First, the SELF-program has been developed within the cyclic framework of the Medical Research Council, which addresses the systematic development, feasibility testing, evaluation and implementation of complex interventions [50]. After the development of the SELF-program, taking into account lessons learned and implications gathered from process evaluations of previous Dutch FFC-based interventions, the SELF-program was feasibility tested in one nursing home ward. The SELF-program was considered feasible in practice and valuable adaptations have been applied prior to the onset of the intended CRT in nursing home care. Another strength, and with that building on implications from previous work, is the incorporation of a more integrated theory of behavior change. More specifically, the SELF-program incorporates a thorough sequential dual behavior change process in which first attempts are made to change the behavior of nurses before they are enabled to improve client’s activity behavior. Although the FFC-philosophy includes behavior change determinants as mechanisms to alter care professionals behavior, recent work questions whether addressing these determinants is sufficient to bring about change. Consequently, it is advocated to include a broader spectrum of behavior change determinants by using integrative behavior change models such as the one addressed in the SELF-program [32]. In addition, we adhere to the suggestions addressed by Lee and colleagues and Verstraten and colleagues to conduct thorough evaluations of FFC-based programs and to use research methods aimed at producing more rigorous burdens of proof [25, 54]. Limitations of the study include the subjective approach of data-collection, i.e., using self-report questionnaires. However, objective measures such as researcher-based observations or videos taken from daily practice may interfere with the client’s and care professionals privacy, for example when examining stimulation behavior and self-reliance in care activities related to personal cleanliness. In line, although it is common in healthcare to collect data from clients with psychogeriatric complaints by their first responsible caregiver – given they have direct and frequent contact to the client in question – we have opted to collect data from all nursing home clients by their primary responsible caregiver. This implies that data from clients with somatic backgrounds will also be collected by their first caregiver, although they might be self-capable to provide the data. However, to address the uniformity of data-collection, and given somatic and psychogeriatric complaints commonly co-occur in Dutch institutionalized care, this approach was considered most feasible. Last, the follow-up period chosen in the CRT is based on practical and financial considerations. However, regarding the outcomes assessment in geriatric clients, i.e., self-reliance in daily activities, it is debatable whether a follow-up period of 6 months is long enough to detect changes. Since the SELF-program assumes that primarily a behavior change is required in care professionals, its translation into adequate changes in day-to-day care practice and subsequently client’s self-reliance may – if any – occur beyond this time period.

The SELF-program offers an advanced and holistic approach to improve activity stimulation behavior of nursing care professionals in long-term care services, and with that optimize levels of self-reliance in ADL in geriatric clients, for a variety of nursing care settings. The results of studies as described in the research protocol are expected to be available from end 2022 onwards. A similar trial is proposed for the home care nursing setting to demonstrate the effect, cost-effectiveness and feasibility of the SELF-program in another long-term care setting.

## Supplementary Information

**Additional file 1.** SPIRIT 2013 Checklist: Recommended items to address in a clinical trial protocol and related documents

**Additional file 2. **Reference: NURS-D-22-00024

## Data Availability

Not applicable; the current paper does not include data and materials.

## Abbreviations

FFC: Function Focused Care
ADL: Activities of Daily Living
MRC: Medical Research Council
ICM: Integrated Change Model
CRT: Cluster-Randomized Trial
GARS: Groningen Activity Restriction Scale
CEA: Cost-effectiveness Analysis
CUA: Cost-utility Analysis
QALY: Quality Adjusted Life Years
