# Supplementary material for: Effect, economic and process-evaluation of a generic function focused care program for long-term care; study protocol of a multicenter cluster–randomized trial
Source: BMC Nurs. 2022 May 19;21:121. doi: 10.1186/s12912-022-00902-5 (PMC9118723; doi:10.1186/s12912-022-00902-5)
Supplement: Supplementary file 2 — Additional file 2. Reference: NURS-D-22-00024 [file 12912_2022_902_MOESM2_ESM.docx]

Reference: NURS-D-22-00024
Title: Effect, Economic and Process-evaluation of a Generic Function Focused Care Program for Long-term Care; Study Protocol of a Multicenter Cluster-Randomized Trial
Journal: BMC Nursing

Through this response letter we would like to thank the editor and reviewer(s) for their constructive and relevant suggestions to the manuscript. Below, we will explain how we handled the identified issues. The remarks of the editor/reviewer are depicted first, broken down to pieces if necessary, and followed by a response in italics. Changes in the manuscript itself are presented using a red font.

Editor Comments:

1. Authors' contributions

-- Please consider the list of authors as it currently stands with reference to our guidelines regarding qualification for authorship (<http://www.biomedcentral.com/submissions/editorial-policies#authorship>).
                                                                    
Currently, the contributions of authors JMG do not automatically qualify them for authorship.  In the section “Authors’ contributions”, please provide further clarifications on their contributions, and see our guidelines for authorship below.

An 'author' is generally considered to be someone who has made substantive intellectual contributions to a published study. Authors are expected to fulfil the criteria below (adapted from McNutt et al., Proceedings of the National Academy of Sciences, Feb 2018, 201715374; DOI: 10.1073/pnas.1715374115; licensed under CC BY 4.0):

Each author is expected to have made substantial contributions to the conception OR design of the work; OR the acquisition, analysis, OR interpretation of data; OR the creation of new software used in the work; OR have drafted the work or substantively revised it

AND to have approved the submitted version (and any substantially modified version that involves the author's contribution to the study);

AND to have agreed both to be personally accountable for the author's own contributions and to ensure that questions related to the accuracy or integrity of any part of the work, even ones in which the author was not personally involved, are appropriately investigated, resolved, and the resolution documented in the literature.

Acquisition of funding, collection of data or general supervision of the research group, alone, does not usually justify authorship.

Anyone who contributed towards the article who does not meet the criteria for authorship can be acknowledged in the ‘Acknowledgements’ section.

**AUTHORS RESPONSE:**

We agree with the comment of the editor. It looks like we forgot to include JMG in the following sentence:

*“ SV, GHW, SM, SZ and JMG were responsible for the detailed design and development of the trial and consequently the reporting of the trial in the current protocol paper. “*

Author JMG was – like all other authors – responsible for the design and development of the trial, including the reporting of the trial protocol in the submitted manuscript.

We have added JMG to the sentence as displayed in red above and in the revised manuscript. Please see page 25 of the revised manuscript, Declarations section, author’s contribution.

For clarity, on page 25 of the revised manuscript, Declarations section, author’s contribution, we have made a minor adaptation to the sentence:

*“ All authors read and approved the final version of the manuscript. “*

Changes in the manuscript are displayed in red.


2. SPIRIT

-- If you do not wish to publish the SPIRIT checklist, then please remove it from the file inventory and include a statement within the manuscript that you adhered to SPIRIT guidelines/methodology.

**AUTHORS RESPONSE:**

We have no objection to publish the SPIRIT checklist which is included as supplementary material in the file inventory. We have added a sentence in the manuscript, please see page 10, Methods section, where we refer to the SPIRIT checklist that it can be found as supplementary material to our trial protocol.

“ The SPIRIT-checklist is available as supplementary material to the trial protocol. “

**----------------------------------------------------------------------------------------------------------------------------------------------------**
